# Functional MRI of working memory and selective attention in vibrotactile frequency discrimination

**DOI:** 10.1186/1471-2202-8-48

**Published:** 2007-07-04

**Authors:** Peter Sörös, Jonathan Marmurek, Fred Tam, Nicole Baker, W Richard Staines, Simon J Graham

**Affiliations:** 1 Imaging Research, Sunnybrook Health Sciences Centre, Toronto, Ontario, Canada; 2 Department of Kinesiology, University of Waterloo, Ontario, Canada; 3 The Rotman Research Institute, Baycrest, Toronto, Ontario, Canada; 4 Department of Medical Biophysics, University of Toronto, Ontario, Canada

## Abstract

**Background:**

Focal lesions of the frontal, parietal and temporal lobe may interfere with tactile working memory and attention. To characterise the neural correlates of intact vibrotactile working memory and attention, functional MRI was conducted in 12 healthy young adults. Participants performed a forced-choice vibrotactile frequency discrimination task, comparing a cue stimulus of fixed frequency to their right thumb with a probe stimulus of identical or higher frequency. To investigate working memory, the time interval between the 2 stimuli was pseudo-randomized (either 2 or 8 s). To investigate selective attention, a distractor stimulus was occasionally presented contralaterally, simultaneous to the probe.

**Results:**

Delayed vibrotactile frequency discrimination, following a probe presented 8 s after the cue in contrast to a probe presented 2 s after the cue, was associated with activation in the bilateral anterior insula and the right inferior parietal cortex. Frequency discrimination under distraction was correlated with activation in the right anterior insula, in the bilateral posterior parietal cortex, and in the right middle temporal gyrus.

**Conclusion:**

These results support the notion that working memory and attention are organised in partly overlapping neural circuits. In contrast to previous reports in the visual or auditory domain, this study emphasises the involvement of the anterior insula in vibrotactile working memory and selective attention.

## Background

Faced with a continuous stream of afferent data, somatosensory processing requires not only the analysis of the properties of tactile stimuli, but also the extraction and encoding of novel, relevant information [1]. The integration of tactile information retrieved from cutaneous afferents, traditionally attributed to the primary (SI) and secondary somatosensory cortices (SII), has been extensively studied [2]. In contrast, the neural basis of tactile working memory and tactile selective attention is less well known. These higher-level cognitive processes are nevertheless crucial for managing many challenges of every-day life. Pulling out a key from a coat pocket in the dark requires, amongst others, exploratory finger movements, attention to tactile information derived from the exploring hand (and not, e.g., from the other hand holding a bag), storage of this information in working memory, and integration of the successively obtained tactile information. Studies on patients with focal lesions suggest that the prefrontal cortex [3,4], right parietal cortex [5] and thalamus [6] are involved in the inhibition of task-irrelevant tactile information. Lesions of the medial temporal lobe, in contrast, have been shown to impair tactile working memory in patients [7].

Building on this small body of literature, the current report describes investigation of the neural substrates of vibrotactile memory and selective attention in healthy volunteers using event-related functional magnetic resonance imaging (fMRI). Participants performed a two-alternative forced choice frequency discrimination task. For this task, participants had to determine whether the second of two consecutive vibratory stimuli was of identical (25 Hz) or higher frequency (> 25 Hz). This task involved not only somatosensory processing, but also other brain functions, including somatosensory working memory, selective attention and planning and execution of a motor response. To assess the effect of working memory, a short (2 s) or a long (8 s) interstimulus interval (ISI) between the first (cue) and the second (probe) stimulus was used, similar to auditory distraction experiments published previously [3]. To test the influence of selective attention, the task was also performed in the presence or absence of a concurrent vibrotactile distractor presented contralaterally. It is hypothesised that delayed vibrotactile frequency discrimination, following a probe presented 8 s after the cue in contrast to a probe presented 2 s after the cue, is associated with mainly dorsolateral prefrontal activation, reflecting working memory [8]. In addition, it is hypothesised that vibrotactile frequency discrimination under distraction mainly involves a right-hemispheric prefrontal-posterior parietal network [9].

## Results

### Behavioural data

The average accuracy of vibrotactile frequency discrimination without distractor was 65% (average response time = 961 ms). In the presence of a simultaneous distractor, the accuracy decreased (47%, F(1,11) = 23.90, p < 0.001) and the response time increased significantly (1369 ms, F(1,5) = 13.08, p < 0.05). Delayed presentation of the probe (ISI = 8 s) significantly reduced the accuracy of vibrotactile discrimination compared to an ISI of 2 s (54% vs. 59%, F(1,11) = 6.29, p < 0.05). The chance level in this forced choice two-alternative decision task was 50%.

### Functional Magnetic Resonance Imaging

**Figure 1:**
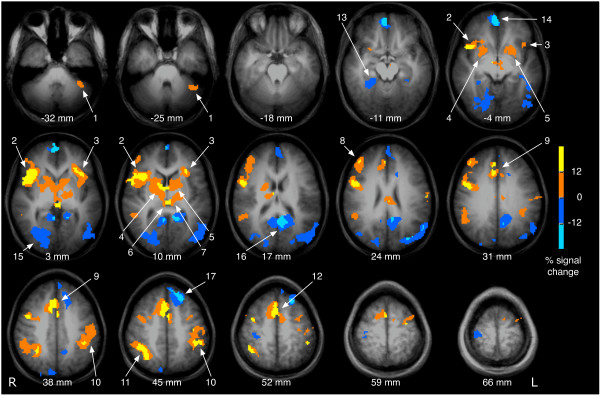
**Brain activation associated with processing of the probe**. The figure shows brain activation and deactivation associated with the processing of the probe (either 25 Hz or higher) across all conditions (clustered activation images with an overall corrected p < 0.05). Activated areas are colour-coded in yellow and red, deactivated areas are displayed in blue. Activation is seen in the left cerebellar hemisphere (1), the bilateral anterior insula (2, 3), the bilateral head of the caudate nucleus and the globus pallidus (4, 5), the bilateral thalamus (6, 7), the right inferior frontal cortex (8), the anterior cingulate cortex (9), the left (contralateral) sensorimotor cortex (10), the right posterior parietal cortex (11) and the supplementary motor area (12). Deactivation was found in the right parahippocampal gyrus (13), the bilateral medial frontal gyrus (14), the right cuneus (15), the bilateral posterior cingulate gyrus (16), the bilateral precuneus (16) and the left superior frontal gyrus (17). Brain images are shown in radiological convention (the right hemisphere is seen on the left side of the image).

Fig. 1 depicts brain activation related to the processing of the probe, regardless of its frequency, in all conditions. Vibrotactile frequency discrimination by button press was associated with widespread activation in cortical and subcortical areas. Activation was seen in the bilateral thalamus (numbers 6 and 7 in Fig. 1), the hand area of the left (contralateral) SI and the left primary motor cortex (10), adjacent to the activation in the postcentral gyrus. Activation was also found in the bilateral anterior insula (2, 3), the anterior cingulate cortex (9), the right posterior parietal cortex (11) and the right inferior frontal cortex (8). In addition, the bilateral basal ganglia (4, 5), in particular the caudate nucleus and the globus pallidus, and the left cerebellar hemisphere (1) were activated. Deactivation was found in the right parahippocampal gyrus (13), the bilateral medial frontal gyrus (14), the right cuneus (15), the bilateral posterior cingulate gyrus (16), the bilateral precuneus (16) and the left superior frontal gyrus (17).

**Figure 2:**
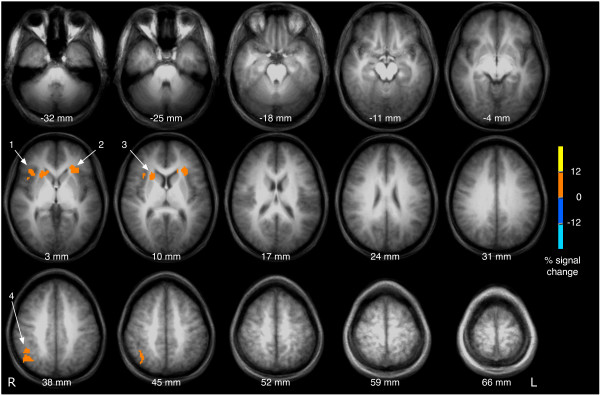
**The effect of a delayed probe**. For this figure, brain activation associated with the processing of the probe was compared between conditions with long and short interstimulus interval (8 s vs. 2 s interval between cue and probe). Clustered activation images with an overall corrected p < 0.05 are shown. The right (1) and left insula (2) as well as the right head of the caudate nucleus and (3) and the right inferior parietal cortex (4) were significantly stronger activated following the probe in trials with an ISI of 8 s compared to trials with an ISI of 2 s. Brain images are shown in radiological convention (the right hemisphere is seen on the left side of the image).

Vibrotactile frequency discrimination with an ISI of 8 s was associated with stronger activation in the bilateral anterior insula (numbers 1 and 2 in Fig. 2), the right caudate nucleus (3) and the right inferior parietal cortex (4) as opposed to the conditions with an ISI of 2 s.

**Figure 3:**
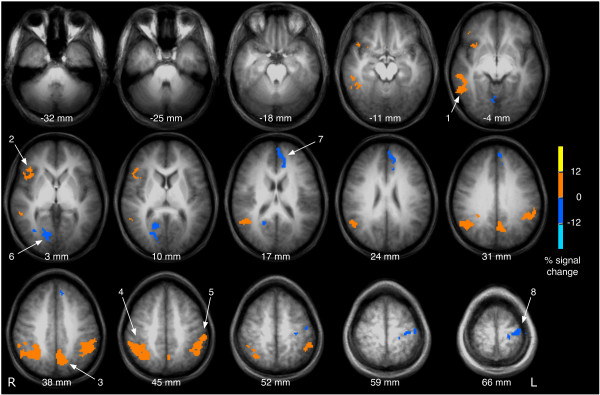
**The effect of a simultaneous distractor**. For this figure, brain activation associated with the processing of the probe was compared between conditions with and without distractor. Areas with significantly stronger activation following the probe with simultaneous distractor compared to frequency discrimination without distractor are colour-coded in yellow and red, areas with less activation are coded in blue (clustered activation images with an overall corrected p < 0.05). Processing of the probe with distractor was associated with increased activity in the right middle temporal gyrus (1), the right anterior insula (2), the left precuneus (3) and the bilateral posterior parietal cortex (4, 5). Deactivation was seen in the right posterior cingulate gyrus (6), the left medial frontal gyrus (7) and the left precentral gyrus (8). Brain images are shown in radiological convention (the right hemisphere is seen on the left side of the image).

Comparing frequency discrimination with and without distractor, the distractor condition was characterised by significantly stronger activation in the right middle temporal gyrus (number 1 in Fig. 3), the right anterior insula (2), the left precuneus (3) and the bilateral posterior parietal cortex (4, 5). Deactivation was seen in the right posterior cingulate gyrus (6), the left medial frontal gyrus (7) and the left precentral gyrus (8).

## Discussion

The results of the present study demonstrate that vibrotactile frequency discrimination is associated with the activation of distributed neural networks, in particular the central somatosensory pathways, the motor system, and the polymodal frontal, parietal and insular cortices (Fig. 1). Under challenge by a simultaneous distractor or a delayed probe, the accuracy of responses decreases in both conditions. The activated brain areas corresponding to distracted or delayed frequency discrimination are partly overlapping, albeit distinct. Bilateral activation of the anterior insula is present in conditions with delayed presentation of the probe (Fig. 2), whereas strong and widespread activation in the bilateral posterior parietal cortex can be found in trials involving a distractor (Fig. 3). The findings of this study are subsequently discussed in light of these sensory and cognitive components. In addition, the significance of the findings is considered for a better understanding of tactile impairments in stroke.

### Vibrotactile frequency discrimination

#### Sensorimotor processing

The chosen vibrotactile stimuli with a frequency around 25 Hz activate primarily Meissner's corpuscles, located in the dermal-epidermal junction of the superficial glabrous skin [10]. Meissner's corpuscles are highly sensitive mechanoreceptors. On the tip of the index finger, perceptual (vibratory detection) thresholds, measured as the peak-to-peak displacement of the vibrating probe, are approximately 10 microns in the frequency range studied here [11,12].

As expected [13-19], vibrotactile frequency discrimination was associated with activation of the somatosensory system, including the contralateral SI and SII, and the bilateral thalamus in the present study (Fig. 1). As the experiment involved button press, cortical and subcortical activation in areas attributed to the planning and execution of voluntary movements was found (contralateral primary motor cortex, the bilateral supplementary motor area, the left ventro-lateral thalamus, the bilateral globus pallidus, and the left cerebellar hemisphere, Fig. 1).

#### Temporal processing

The present results demonstrate that vibrotactile frequency discrimination is associated with a robust activation of the bilateral anterior insula (Fig. 1). The insula is a highly multimodal area which integrates information from several distinct regions of the brain [20]. Converging evidence shows that the insula is functionally separated along the anterior-posterior axis. In the sensory domain, a direct comparison of painful thermal and innocuous tactile stimulation using fMRI revealed that the perception of pain was associated with activation of the anterior part, while the perception of tactile stimuli was associated with activation of the posterior part of the insula [21,22]. In addition to the perception of pain, the anterior insula is involved in the processing of innocuous, but unpleasant stimuli (vibratory stimulation of the teeth [23]) as well as of visceral [24] and gustatory afferents [25].

For the interpretation of the present findings, however, the involvement of the anterior insular cortex for the processing of temporal aspects of stimuli (e.g., pitch [26]) and for the preparation of highly time-dependent movements (e.g., singing and speaking [27,28]) might be important. In addition, lesion studies in cats demonstrated that the temporal operculum and the underlying insular cortex are involved in vibrotactile temporal pattern discrimination [29]. Based on these reports, it is hypothesised that the bilateral anterior insula is involved in the analysis of the temporal aspects of vibrotactile stimuli. In support of this hypothesis, passive vibrotactile stimulation without discrimination between stimuli of different frequency [13-18,30] did not activate the anterior insula.

#### Default mode network

Compared with baseline, vibrotactile frequency discrimination was associated with deactivation in the frontal cortex (medial and superior frontal gyrus), the cuneus, the precuneus, the parahippocampal area and the posterior cingulate gyrus (Fig 3). These areas probably reflect a widespread neuronal network that is consistently activated during rest or during less demanding tasks, termed the default mode network [31]. In a highly demanding task, such as the frequency discrimination task used here, the putative default mode network is expected to be deactivated when comparing task vs. rest. This interpretation is corroborated by the results of several investigations on cognitive and sensory processing. In a study on visual and auditory processing, the default mode network, similar to the deactivated areas in the present study, was supposed to consist of medial frontal areas, the posterior cingulate, the hippocampus and the parahippocampus [32].

### Vibrotactile working memory

In the present study, the function of vibrotactile working memory was selectively probed by varying the interval between the first and the second stimulus. Comparing the conditions with long vs. short ISI uncovered activation of the bilateral anterior insula, the right head of the caudate nucleus and the right inferior parietal cortex (Fig. 2).

Working memory refers to the ability to maintain and manipulate information temporarily and has been widely investigated in the visuo-spatial, auditory, and verbal domains. Traditionally, the prefrontal cortex has been regarded as an important neural correlate of working memory [9,33]. In the present study, the inferior frontal cortex is activated in the analysis of all events (Fig. 1), but is not differentially active when increasing the ISI from 2 s to 8 s (Fig. 2), probably due to the lower statistical power of the subgroup of events.

Knowledge about the neural correlates of vibrotactile working memory, however, is limited. There is evidence that the neural correlates of tactile working memory include areas involved in the sensory processing of tactile stimuli [34]. Using vibrotactile frequency discrimination tasks similar to the 2 s ISI task employed here, but without conditions with longer ISI, single-cell recordings in primates [35] and transcranial magnetic stimulation in humans [36] demonstrated that vibrotactile memory traces are maintained in SI. The present study did not reveal stronger activation in SI or SII when increasing the ISI from 2 s to 8 s.

The data presented here suggest that areas found active in previous studies on cognitive and sensory working memory are also involved in vibrotactile memory. An investigation of working memory using a sequential letter (n-back) task and fMRI revealed activation in the bilateral inferior parietal cortex (supramarginal gyrus) and the bilateral frontal operculum, along with activation of the bilateral inferior frontal gyrus and other areas [37]. The results here imply that the bilateral anterior insula is not only involved in analysing temporal properties, but also subserves as a neural correlate of working memory for temporally complex stimuli. Because of the dense connections between the prefrontal cortex and the caudate nucleus, the caudate is thought to be involved in working memory as well [38].

### Selective attention

The effect of selective attention was investigated by simultaneously applying a distractor stimulus to the contralateral thumb in 25% of the epochs. Selective attention is often regarded as a cognitive process selecting a subset of information for further processing. Vibrotactile distraction through a contralateral vibrotactile stimulus is associated, on the behavioural level, with a decrease of response accuracy and, on the neuronal level, with an increase of activation in the bilateral posterior parietal cortex, the right anterior insula, and the middle temporal gyrus (Fig. 3). The observed decrease of accuracy confirmed a previous pure behavioural experiment [39].

Converging lines of evidence support the notion that a widespread, mainly right-hemispheric fronto-parietal network is involved in selective attention. In the tactile domain, the posterior parietal cortex has been found active during tasks involving selective attention using fMRI [40] and somatosensory evoked potentials [41]. The present results, showing bilateral posterior parietal activation, corroborate these findings. Moreover, processing of a sensory stimulus is enhanced by selective attention to the stimulus. This has been shown in the visual [42], auditory [43], and somatosensory domain [44]. The posterior parietal cortex, however, contains neurons with bilateral receptive fields [45]. Activation of those neurons by bilateral vibrotactile stimulation during the distractor conditions might contribute to the bilateral posterior parietal activation seen here.

Increased activity in the right anterior insula under distraction is hypothesised to represent increased analysis of temporal stimulus properties as discussed above. Activation of the right middle temporal gyrus has previously been related to language functions, such as semantic processing [46] and processing of affective prosody [47]. In addition to these reports, the present study indicates that the right middle temporal gyrus is also involved in selective attention to vibrotactile stimuli. This interpretation is supported by a high-density EEG study in which the bilateral middle temporal gyri were involved in selective spatial attention [41].

Comparing the brain activity between delayed (Fig. 2) and distracted (Fig. 3) frequency discrimination, the right anterior insula and the posterior parietal cortex are active in both conditions. This finding supports the idea that working memory and attention are represented by distributed, partly overlapping neuronal networks [48,49].

### Relevance for tactile deficits in stroke

Tactile working memory and selective attention are important cognitive processes for complex sensory processing in humans, beyond the experimental situation of vibrotactile frequency discrimination. Tactile working memory and selective attention are crucial for object recognition or manipulation without visual feedback or in situations where somatosensory information from different body parts (walking, bimanual operations) or information from different sensory modalities compete.

For the present study, a forced-choice vibrotactile frequency discrimination task has been developed that is also applicable to future investigations of stroke patients. Deficits of somatosensory processing are frequent symptoms of stroke and often have far-reaching consequences for the independence and the quality of life of stroke patients [50]. Although impaired motor or language functions often impose a higher burden on the individual patient, the integrity of tactile functions is an important predictor for the long-term recovery of stroke patients [51-53]. Many functional abilities, such as the control of grip force [54], and gait [55] can be affected by deficits of tactile processing.

## Conclusion

The present study demonstrates that vibrotactile frequency discrimination is associated with the activation of distributed neural circuits including the somatomotor system, and polymodal frontal, parietal, and insular areas. Brain networks involved in distracted or delayed frequency discrimination are partly overlapping, albeit distinct. Bilateral activation of the anterior insula is present in conditions with delayed presentation of the probe, while strong and widespread activation in the bilateral posterior parietal cortex can be found in trials involving a distractor. The novel findings of this study on healthy adults and the partial differences between our findings and the results of lesion studies warrant further investigations of brain activity associated with impaired somatosensory processing in stroke patients.

## Methods

### Participants

Twelve healthy, right-handed volunteers (6 men, 6 women, mean age 22 years) participated in the study. The study was approved by the Research Ethics Board of the Sunnybrook Health Sciences Centre. Informed consent for participation in the project was obtained from all participants according to the Declaration of Helsinki.

### Stimulation

**Figure 4:**
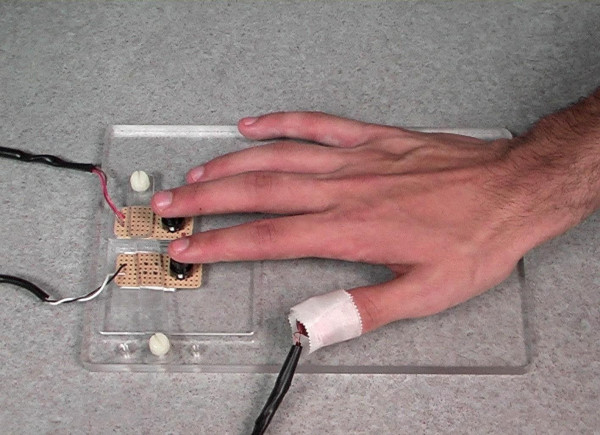
**Experimental setup**. A magnetomechanical vibrotactile device is taped to the right thumb. The index and the middle finger rested on a two-button response pad. The arms were extended during the measurement. Pressure points were avoided using foam pads.

Magnetomechanical vibrotactile devices (MVDs) were used for stimulation as described previously [15]. These MVDs allow precise control of the frequency and amplitude of vibrotactile stimulation through custom software. The MVDs used here were able to generate frequencies of at least 100 Hz. The use of higher frequencies is possible but is restricted by the stability of the mount and the size of the coil. Prior to the experiment, MVDs were taped to the palmar surface of the distal phalanges of both thumbs (Fig. 4). Responses were made with the right index or middle finger using a two-button response pad, also built within the laboratory (Fig. 4). Practice trials were performed inside the scanner, during the structural MRI and before the fMRI experiment, to assess individual perceptual thresholds for the 25 Hz vibrotactile stimulus. Perceptual thresholds were defined as the smallest stimulation amplitudes that could be reliably identified and were determined using the alternating staircase method. In the actual experiment, stimulation amplitudes were set to 700% of the individual perceptual threshold. In addition, the vibrotactile discrimination threshold f was assessed, where f is the minimal difference between cue (25 Hz) and probe (25 + f Hz) that could be detected with 80% accuracy. The discrimination threshold f was determined by assuming a normal distribution of the frequency differences that were detected correctly (f_corr_) during the iterative procedure, such that Z = (mean(f_corr_) -f)/standard deviation(f_corr_) = 0.84 for 80% success. In all cases, the assumed frequency f was × 10 Hz. During the fMRI experiment, however, participants performed lesswas accurate than predicted by this formula (65% accuracy on average). As we tried to minimize the time participants had to stay in the magnet before the beginning of the fMRI experiment, thresholds were determined during the structural MRI (scan time: 8 min) and not in a separate test run. The number of iterations achieved during this time might have been too low for an accurate estimation of discrimination thresholds. Moreover, participants might have developed fatigue or loss of concentration during the preparation for the fMRI experiment (15 – 20 min including structural MRI) resulting in decreased discrimination accuracy later on.

**Figure 5:**
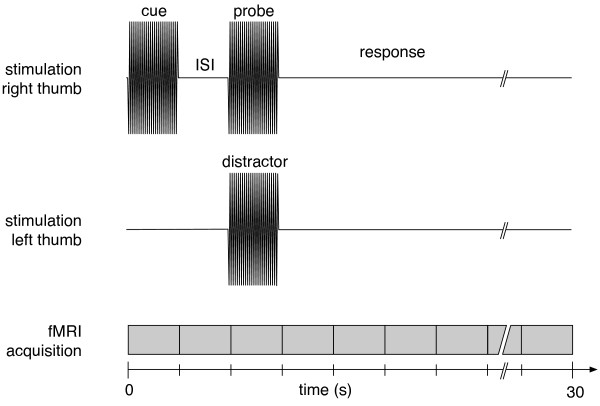
**Illustration of the experiment**. Upper graph: A vibrotactile stimulus (frequency: 25 Hz; duration: 2 s) was delivered to the right thumb (cue) followed by an analogous probe of either identical frequency or higher frequency (25 Hz + individual discrimination threshold f). The interstimulus interval (ISI) was either 2 s (as illustrated here) or 8 s. Lower graph: In 25% of trials the probe was paired with a distractor to the left thumb. The stimulation parameters of the distractor were identical to those of the cue. Functional MRI data were obtained continously. Every 2 s, a brain volume consisting of 26 axial was acquired, starting with the beginning of each trial.

Fig. 5 provides a schematic illustration of the experiment. Eight different stimulation conditions were applied in a pseudo-randomised order (Tab. 1). Each condition started with a 25 Hz cue stimulus followed by a probe stimulus, both delivered to the right hand. All vibrotactile stimuli were presented for 2 s. For all events, the time between the onset of two consecutive cue stimuli was 30 s. The frequency of the probe was either 25 Hz or higher (25 + f Hz, with equal (50%) probability). Participants were asked to decide if the frequency of the probe was identical to or different than the cue in a forced choice two-alternative decision task. Participants were instructed to respond as quickly and as accurately as possible after the offset of the probe. Vibrotactile stimulation and the recording of behavioural responses were controlled by a PC running LabVIEW (National Instruments, Austin, TX, USA). To assess the influence of the interstimulus interval (ISI) between cue and probe, the ISI was either 2 s or 8 s (probability: 50% each). To assess the influence of a distractor, in 25% of all trials an analogous vibrotactile stimulus was delivered to the left thumb simultaneously to the probe. The experiment consisted of four runs with 16 trials per run (64 trials in total). The acquisition of all functional images required 32 min. The total scan time, including the anatomical scan and the initial behavioural testing, was approximately 1 h.

**Table 1:** Outline of the applied stimulus conditions.

Condition	Frequency of probe	ISI	Distractor	Probability
1	25 Hz	2 s	no	combined: 75%
2	25 Hz	8 s	no	
3	25 + f Hz	2 s	no	
4	25 + f Hz	8 s	no	

5	25 Hz	2 s	yes	combined: 25%
6	25 Hz	8 s	yes	
7	25 + f Hz	2 s	yes	
8	25 + f Hz	8 s	yes	

Note: Stimulus conditions were applied in pseudo-randomised order. The frequencies of the cue and the distractor stimuli were always 25 Hz. Cue and probe stimuli were delivered to the right hand, distractor stimuli to the left hand. f denotes the minimal difference between cue and probe stimuli that could be detected with 80% accuracy.

Accuracy (% correct) was calculated for each condition and subjected to repeated measures ANOVA (SPSS, SPSS Inc., Chicago, IL, USA) with a full within-participants Distractor × Delay × Probe model. Mean response times (for correct responses only) were analysed similarly, using a maximum likelihood algorithm to account for missing data (BMDP-5V, BMDP Statistical Software, Los Angeles, CA, USA).

### Magnetic Resonance Imaging

Structural and event-related fMRI was conducted using a Signa VH/i 3.0 T scanner and quadrature birdcage head coil (GE Healthcare, Waukesha, WI, USA). A single-shot spiral sequence was used for blood oxygenation level-dependent fMRI (TR/TE/flip = 2000 ms/30 ms/70°, matrix 64 × 64, FoV 20 cm, and 26 axial slices 5 mm thick) [56]. The reconstructed fMRI data were processed in AFNI [57]. After motion correction, general linear modeling (GLM) was used to estimate the system response to the probe in each condition. Percent signal change maps were formed from the integrated responses, normalised to Talairach-Tournoux space, and blurred with a 5 mm FWHM Gaussian filter. Linear contrasts of these maps for all participants underwent one-way t-tests to generate group maps for all Distractor × Delay × Probe main effects and interactions. For the analysis of the probe in all conditions, the group maps were thresholded at a voxelwise p < 0.005 with a minimum cluster size of 0.87 ml within a radius of 2 mm which amounts to an overall (corrected) p < 0.05 [58]. For the analysis of the effects of delay and distractor, the group maps were thresholded at a voxelwise p < 0.01 with a minimum cluster size of 1.46 ml within a 5 mm radius which amounts to an overall (corrected) p < 0.05.

## Abbreviations

fMRI functional magnetic resonance imaging

ISI interstimulus interval

MVD magnetomechanical vibrotactile device

SI primary somatosensory cortex

SII secondary somatosensory cortex

## Authors' contributions

SJG and WRS designed the experimental setup of the study. NB developed the stimulation device and implemented the measurement of vibrotactile discrimination thresholds. JM and FT acquired the data and performed the statistical analyses. PS drafted the manuscript, and the other authors contributed substantially to it. All authors read and approved the final manuscript.
